# Financial development and economic growth in Sub-Saharan Africa using system GMM analysis

**DOI:** 10.1371/journal.pone.0349118

**Published:** 2026-06-04

**Authors:** Temesgen Yaekob Ergano, Sure Pulla Rao

**Affiliations:** 1 Department of Economics, College of Business and Economics, Wachemo University, Hoss’na, Ethiopia; 2 Head of the Department of Economics, College of Arts & Commerce, Andhra University, Visakhapatnam, Andhra Pradesh, India; Gulu University, UGANDA

## Abstract

The study on financial development and economic growth in Sub-Saharan Africa utilises System GMM analysis to investigate the relationship between financial development indicators and regional economic performance. The research findings reveal significant impacts of various financial indicators on economic growth, such as the positive influence of bank liquid reserves on bank assets ratio (R/A), trade openness, and the broad money to total reserves ratio (M/R) on the economic growth of Sub-Saharan Africa. Additionally, the study highlights the negative impact of Credit extended to the private sector by banks within the country (D_bank) on economic development, emphasising the importance of prudent credit allocation to avoid over-indebtedness and financial crises. These results provide valuable insights for policymakers aiming to foster sustainable economic growth in the region by leveraging financial development effectively.

## 1. Introduction

Financial development is defined as the accumulation of financial assets at a faster rate than non-financial assets, and it involves the evolution of financial instruments, markets, and intermediaries to reduce costs and improve the provision of financial services. Economic growth, on the other hand, is the long-term increase in a country’s Gross Domestic Product (GDP), reflecting the value added produced by all firms operating within the country. Financial development is critical to economic growth and resilience, especially in emerging and developing economies, as it facilitates information sharing, efficient resource allocation, and risk management. It also promotes financial stability by creating deep and liquid financial systems [1].

The relationship between financial development and economic growth volatility is complex and has been the subject of various studies. Research suggests that a well-developed financial system can smooth economic growth by reducing financial constraints that cause business cycles to propagate. Others argue that the effects of financial development on

Growth depends on real and monetary shocks, credit supply, and the stage of financial development [2]

In the context of Sub-Saharan Africa, the financial sectors are still in the early stages of development and are multiplying. This rapid growth, combined with vulnerabilities such as limited regulatory capacity, poses risks to financial system stability. Despite the infrequency of systemic banking crises on the continent, the fast credit growth in many economies calls for caution and the need for robust and countercyclical regulation of the African financial systems [3].

Research on the impact of financial development on economic growth has yielded mixed findings. Some studies argue that financial intermediaries may hinder economic growth, while others suggest that financial development can lead to macroeconomic instability. Recent studies have identified a nonlinear relationship between economic indicators, such as the monetary reserve ratio, openness to trade, bank reserve ratio, and monetary base or money supply, with economic growth indicating a positive correlation.

This study introduces an innovative methodology to explore the connection between financial development and economic growth. It utilises panel regression with pool analysis and fixed effects, complemented by a combination of the Generalised Method of Moments (GMM) for model selection. Additionally, the research incorporates sensitivity analysis and dynamic panel regression analysis to enhance the robustness and reliability of the results. The application of the System Generalised Moments method (SGMM) enables a thorough investigation of the robust relationship between financial development and economic growth, particularly in the context of Sub-Saharan African countries

## 2. Literature review

### 2.1. Theoretical literature review

The relationship between financial sector development and economic growth has been the subject of extensive theoretical exploration and empirical investigation globally. The debate surrounding this nexus traces back to the early twentieth century, with scholars like Schumpeter (1911) questioning whether financial development influences economic growth positively or negatively. The discourse has evolved to examine the impact of financial depth and efficiency on economic performance [4]

Financial depth, measured by indicators like broad money (M2/GDP) and financial system deposits (Deposits/GDP), reflects the capacity of the financial system to support economic activities. On the other hand, financial efficiency assesses how effectively resources are allocated within the financial system to enhance economic growth.

The theoretical framework often posits that a well-developed financial sector can stimulate economic growth by facilitating investment, savings mobilisation, and resource allocation. Scholars like [5] and [6] have found evidence supporting a positive relationship between financial development and economic growth in various countries. Conversely, studies by [7], and [8]) have highlighted contrasting outcomes, including negative or no significant impact of financial development on economic growth.

Moreover, theoretical models adapted from De Gregorio and [3] provide a framework to analyse the empirical link between financial development indicators (such as bank deposits, private investment, and government expenditure) and economic growth. These models incorporate endogenous growth theories to elucidate how financial variables influence long-term economic performance.

### 2.2. Empirical literature review

Financial sector development and economic growth reveal contrasting findings from various studies across different countries. [9] conducted a cross-country regression analysis involving 49 countries, suggesting that financial development acts as an anti-growth factor. In contrast,

[6] found a strong correlation between economic growth and financial sector development in 80 countries. [1] studied the United Arab Emirates using an ARDL approach and identified a negative relationship between economic growth and financial sector development and bidirectional Causality between the two factors.

Moreover, [10] analysed 75 countries through time-series analysis and concluded no significant relationship exists between economic growth and financial sector development. On the other hand, [11] examined China using a generalized method of moment system estimation and found that financial sector development positively affected productivity growth. Yıldırım et al. (2013) focused on emerging European economies and discovered unidirectional Causality from economic growth to financial sector development.

In the context of Cameroon, Tabi et al. (2011) utilised the Johansen method of co-integration analysis and reported a positive effect of financial development on economic growth. [2]

Studied 21 Sub-Saharan African countries using the dynamic panel GMM technique and established a positive link between financial development and economic growth. [12]investigated SSA’s financial development and economic growth, finding a positive impact in both countries when different estimation methods were used.

The research by [13]in Cameroon and South Africa using VECM revealed a long-running relationship between bank deposits and economic growth in Cameroon. At the same time, South Africa showed an independent relationship with different estimation methods. These studies highlight the complex relationship between financial sector development and economic growth, showcasing varying impacts based on methodologies, countries, and specific characteristics.

This literature review underscores the importance of considering diverse perspectives to understand the nuanced effects of financial development on economic growth across different regions globally.

## 3. Methodology and data

This section discusses the theoretical framework and estimation techniques used in the study. It also provides information on the definition of variables and sources of data used in the analysis.

### 3.1 Theoretical framework

The study’s theoretical foundation is rooted in the [14] long-run growth model, which elucidates long-term economic expansion through population growth, capital accumulation, and technological advancements. Employing the Cobb-Douglas production function, the model is encapsulated in equation (1)


$$\begin{array}{c} Y(t)\;={K(t)\;(A(t)L(t))}^{1-\alpha }\; \end{array}$$ (1)

Where 0<α< 1, α is the share of labour in output, t denotes time, Y(t) denotes total production, K(t) is capital, L(t) is labour, A(t) represents labour-augmenting technology and A(t)L(t) denotes effective labour.

The expression for the performance of capital stock per effective worker, denoted as **k** over time, is given by the following equation:


$$\begin{array}{c} k\left(t\right)=sk{\left(t\right)}^{\alpha }-\left(n+g+\delta \right)\;k\;(t) \end{array}$$ (2)

Where s is the savings rate, g is technological progress, n is population growth rate, and 𝛿 is depreciation. This suggests that k(t) gets closer to the steady state value of k* defined by equation 3:


$$\begin{array}{c} ss\;\left(k^{*}\right)\;\left(t\right)=\left(n+g+\delta \right)k^{*}\left(t\right) \end{array}$$ (3)

The steady-state capital per effective worker can be expressed as Equation 4


$$\begin{array}{c} K^{*}={\left(\frac{s}{n+g=s}\right)}^{\frac{\text{1}}{\text{1}-\alpha }} \end{array}$$ (4)

With equation (4), the units of effective labour **A(t)L(t)** and capital stock **K(t)** are known to be growing at a rate of **(n + g)**. Simultaneously, **Y(t)** also increases at the same rate due to the constant returns to scale that the theory assumes. Now, let’s weave these threads together. Substituting equation (4) into the steady-state output per effective worker, we unveil a poetic equation.


$$\begin{array}{c} y^{*}={\left(\frac{s}{n+g+s}\right)}^{\frac{\text{1}}{\text{1}-\alpha }} \end{array}$$ (5)

Taking the natural logarithm of equation 5, we obtain equation 6:


$$\begin{array}{c} ny^{*}=\frac{\alpha }{\text{1}-\alpha }lns-\frac{\alpha }{\text{1}-\alpha }\text{In}(n+g+s) \end{array}$$ (6)

This indicates that the growth in steady-state output per effective worker, denoted as y*, is dependent on the savings rate (s) and positively correlates with the steady-state output per effective worker. Consequently, an increase in s would lead to enhanced output (economic growth) by boosting investments. However, technological progress (g), population growth rate (n), and the rate of depreciation (𝛿) are inversely associated with output growth and capital per effective worker.

Financial development can influence economic growth in several ways. By increasing the capital stock, financial development facilitates growth. Proxies for development are Bank liquid reserves to bank assets ratio (**R/A**), Monetary growth(**M_G**), and Broad money to total reserves ratio(**M/R**), along with openness to trade and domestic credit.

To the private sector by banks(D_bank), Domestic credit to the private sector(D_all), GDP deflator GDP per capita and CPI as a proxy of inflation contribute to the physical capital (K). Financial development not only supplements savings but also augments the capital stocks of countries with insufficient savings for investment projects, thereby helping to bridge the resource gap

### 3.2. Estimation technique and empirical model various

#### 3.2.1. Panel analysis.

For the panel dataset, we employ pooled OLS (POLS), a fixed effect specification (FE), and the dynamic panel Arellano-Bond estimator (AB), where the instruments are the lags of the explanatory variables (in levels and differences). The main motivation for the use of AB is the presence of independent variables that may not be strictly exogenous, such as the financial development variables, and to present a dynamic specification, avoiding “dynamic panel bias”

#### 3.2.2. GMM specification.

We use dynamic panel data estimators (difference GMM and system GMM) developed to properly test the hypothesis that remittances can affect economic growth (Arellano, M., and S. Bond., 1991), (Arellano, 1995) and (Blundell, R., and S. Bond., 1998) to develop our growth model. The difference GMM estimator is calculated by transforming all regressors, typically by differencing, whereas the system GMM model is composed of stacked regressions in levels and differences. The baseline regression of our GMM specification is the following


$$\begin{array}{c} RGDPit=\beta \text{0}+\beta \text{1}RGDPit-\text{1}+\beta \text{2}FDit+\beta \sum_{i=\text{1}}^{n}Xit+\lambda i+\mu t+\varepsilon it \end{array}$$ (7)

Whereas RGDP proxy for growth, FD financial development peroxide by (Monetary Sector credit to private sector(MSP) (% GDP), ODA, Domestic credit to private sector by banks(BS) Domestic credit to private sector(CPS) (% of GDP), X represents a vector of conditioning information that controls for other factors associated with economic growth, λi is an unobserved country-specific effect, ut is time-specific effect and εit is the error term

Its nonlinear specification form becomes


$$\begin{array}{c} RGDPit=\beta \text{0}+\beta \text{1}RGDP,t-\text{1}+\beta \text{2}FDit+\beta \text{3}qit+\beta \text{4}(FDit*qit)+\text{¥}\sum_{i=\text{1}}^{n}Xit+\lambda i+\mu t+it \end{array}$$ (8)

The GMM specifications include the same covariates as before, while the interaction term demonstrates a change in the behaviour of financial development effects after some structural breaks.

While the lagged levels of the exogenous variables are used as instruments in the difference GMM model, the GMM system estimator uses lagged differences of the explanatory variables as instruments for equations in levels, as well as lagged levels of the explanatory variables as instruments for equations in first differences. These models have been widely used to address the endogeneity issue that arises in the estimation of growth regressions using panel data. (Arellano, 1995); (Blundell, R., and S. Bond, 1998). They also account for biases caused by country-specific effects or the presence of initial GDP in the growth covariates. Finally, as discussed in the preceding studies, GMM avoids simultaneity and reverse causality issues. To ensure the consistency of the GMM estimators, two diagnostic tests are run: The Hansen test for over-identifying restrictions, where the null hypothesis is that the instruments are not correlated with the residuals, and Arellano-Bond’s test for second-order correlation in the first differenced residuals.

### 3.3 Data and measurement

**Table 1 *provides detailed descriptions of the variable***s, their expected signs, and why we included them in our study. Our research focuses on 47 countries in Sub-Saharan Africa from 1998 to 2023. These countries were chosen based on the availability of Financial development data during this period. We gathered data on various indicators such as OPP, BRR, R/A, and M/R, along with GDPD, D_all, and D_bank, M_G, and CPI as a proxy for inflation from the World Development Indicators (WDI) database (World Bank,2021). To analyse the data, we utilized the Stata 15.0 statistical package. By using the robust command Please note that all data presented in this analysis has been sourced from the World Bank indicators (WB,2024) and the International Monetary Fund (IMF,2002)

**Table 1:** Variable definition and expected signs.

Variable	Definition	Expected sign
real gross domestic product:	The total value of all goods and services produced in an economy, adjusted for inflation.	+
The ratio of bank liquid reserves to bank assets (R/A)	The ratio of bank liquid reserves to bank assets (R/A)ratio of a bank’s total assets to its liquid reserves	+/-
Broad Money-to-Total Reserves Ratio(M/R)	Compares total broad money to total reserves held by banks.	+/-
Broad Money Growth(M_G):	The rate at which broad money supply increases over time	+
Private Sector Domestic Credit by(D_B)	Banks (D_B) The quantity of credit that banks provide to the private sector	+
Index of Consumer Prices (CPI)	calculates the average change in prices that customers pay for a variety of goods and services.	+/_
Private Sector Domestic Credit (D_all)	total amount of domestic credit that is accessible to the private sector from sources other than banks.	: +
GDPC stands for gross domestic product per capita	GDP is divided by population, indicating the average economic output per person.	+
GDP Deflator(GDPD)	Measures price inflation within the economy, reflecting changes in price levels over time.	+
**Openness to Trade (OPP)**	Measures the extent of international trade relative to GDP	+/_

### 3.4. An Overview of Sub-Saharan Africa’s Financial Development (1998_2022)

Between 1998 and 2022, Sub-Saharan Africa’s (SSA) financial development environment saw significant changes brought about by improved access to financial services, technological advancements, and policy reforms. This discussion will explore these transformations through the lenses of relevant proxies such as the Ratio of Assets (R/A), Monetary Growth (M_G), and Money to Reserve (M/R).

#### 3.4.1. Money Growth in Sub-Saharan Africa Over the Last Decades (1998_2022).

**Fig 1:**
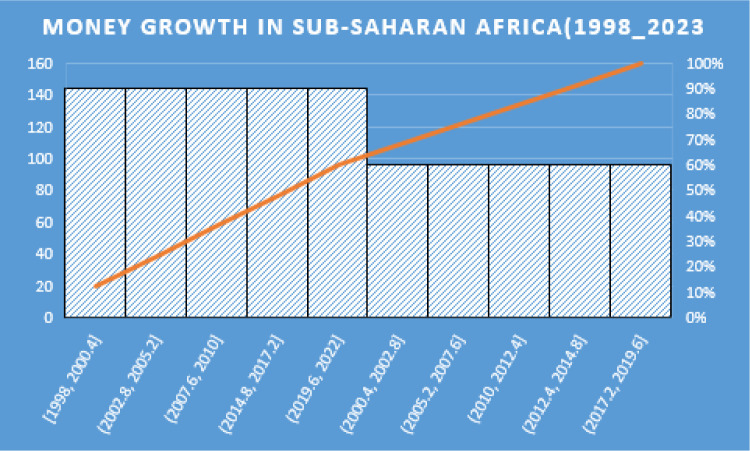
Money growth in Sub-Saharan Africa (1998_2023). Source: Own Computation.

Fig 1, Money growth in Sub-Saharan Africa (1998_2022) shows that the timeframe spanning from 1998 to 2023 has witnessed significant changes in money growth across Sub-Saharan Africa (SSA), influenced by a blend of economic reforms, technological progress, and external circumstances. The increase in domestic credit given to the private sector, which went from 61.23% of GDP in 2000 to 65.58% in 2019, has been a defining feature of monetary growth in Sub-Saharan Africa. This pattern points to a growing banking industry that is steadily improving its capacity to raise capital for investment.(Ustarz & Fanta, 2021a).

Sub-Saharan Africa’s economies have performed unevenly, despite advancements in money growth and financial development. Burundi and Zimbabwe have struggled with stagnation or decline, while Ethiopia and Angola have seen strong growth rates. The average annual growth rate of GDP per capita for SSA has fluctuated significantly over the decades, highlighting disparities in economic performance among nations(Guru & Yadav, 2019)

#### 3.4.2. Summary of Money to reserve ratio in Sub-Saharan Africa (1998_2022).

**Fig 2:**
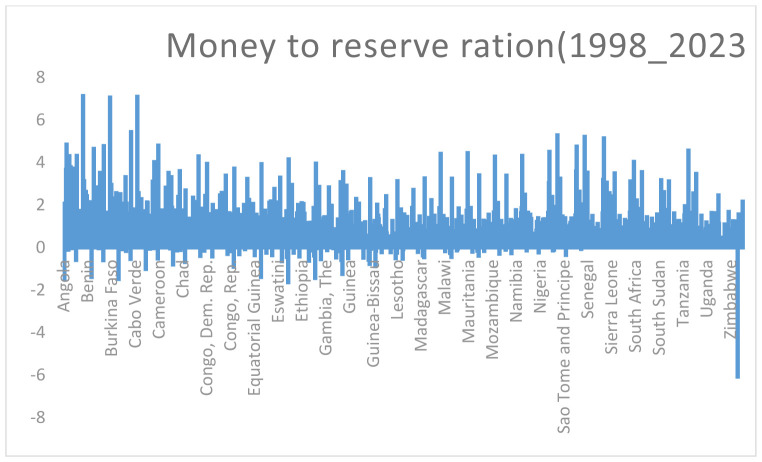
Liquid Money to reserve asset ratio of Sub-Saharan Africa. Source: Own Computation.

Fig 2, money-to-reserve ratio summary for Sub-Saharan Africa (1998 _ 2022) reveals significant changes in monetary policy and their effects on the region’s financial stability. Countries with relatively high money-to-reserve ratios include Benin, Nigeria, São Tomé and Príncipe, Cabo Verde, and South Africa, as shown in, Fig 2, On the other hand, the Democratic Republic of the Congo (DRC), Zimbabwe, and Lesotho have the lowest money-to-reserve ratios in the region.

Countries with High Money-to-Reserve Ratios: Benin and Cabo Verde have been actively implementing reforms to strengthen their financial systems, enhancing lending capabilities. As of November 2024, Nigeria stands out with a cash reserve ratio set at 50%, reflecting the central bank’s strategic efforts to manage liquidity while facilitating substantial lending activities.

Countries with Low Money-to-Reserve Ratios: Conversely, nations such as Zimbabwe, which has endured significant economic turmoil and hyperinflation, face challenges associated with low money-to-reserve ratios. This situation reflects a lack of confidence in the banking system and severely limits lending capabilities. Similarly, Lesotho and the Democratic Republic of Congo (DRC) also display low ratios, indicating underlying economic instability or restrictive monetary policies that hinder banks’ ability to extend credit.

In Lesotho and the DRC, low money-to-reserve ratios may be symptomatic of broader economic issues, including political instability and limited access to financial services. These factors contribute to a constrained lending environment, stifling economic growth and development.

#### 3.4.3. An overview of Sub-Saharan Africa’s banks’ liquid reserves to assets ratio (1998–2022).

**Fig 3:**
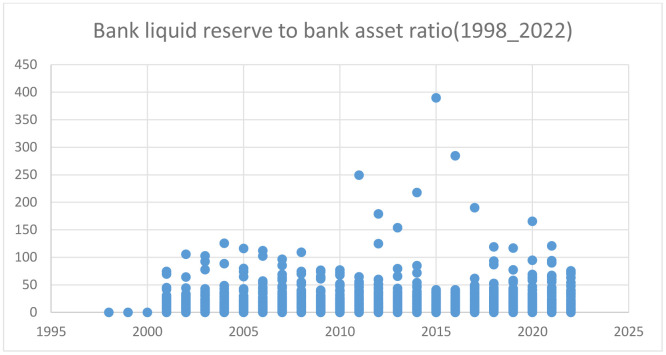
Bank liquid reserve to bank asset ratio of Sub-Saharan Africa. Source: Own Computation.

Fig 3, Bank liquid reserve to bank asset ratio of Sub-Saharan Africa shows that the ratio of liquid reserves to total bank assets is essential to a bank’s liquidity and overall financial well-being. This ratio indicates the share of a bank’s liquid assets, encompassing cash and deposits held with central banks, with its total asset base. In Sub-Saharan Africa (SSA), this indicator has exhibited considerable fluctuations between 1998 and 2023, shaped by macroeconomic factors, regulatory developments, and the stability of the regional financial environment.

Between 1998 and 2003, Sub-Saharan Africa’s (SSA) average liquid reserves ratio was comparatively low, with significant variations between nations. According to “Bank. Sub-Saharan Africa” (2013), the average reserve ratio in 2004 was 11.3%, with extremes ranging from 0% in the Central African Republic to almost 50% in nations like Mauritius.

From 1998 to 2022, the bank liquid reserves to bank assets ratio across Sub-Saharan Africa has shown diverse trends influenced by various factors such as regulatory frameworks, economic conditions, and institutional quality. As the region continues to evolve economically and politically, maintaining adequate liquidity will be critical for ensuring financial stability and fostering sustainable growth within the banking sector.

## 4. Results and discussions

### 4.1. Descriptive analysis

The summary statistics from Table 2 provide valuable insights for the countries under consideration:

**Table 2:** Summary of Statistics.

Variable |	Obs	Mean	Std. Dev.Min		Max
**+**					
**RGDP |**	**1,152**	**22.92435**	**1.480331**	**18.77475**	**27.00616**
**R/A |**	**920**	**2.878732**	**.8249815**	**.4937103**	**5.966429**
**OPP |**	**899**	**.6994543**	**.4374776**	**.0631788**	**3.650533**
**GDPC|**	**1,152**	**11.56909**	**2.397545**	**4.526336**	**16.58105**
**M_g |**	**1,089**	**3.232526**	**.6595083**	**−3.344119**	**5.174956**
**+**					
**INF |**	**1,018**	**1.820943**	**1.223747**	**−4.696737**	**7.874786**
**GDPD |**	**1,152**	**4.472884**	**1.002485**	**−1.664606**	**11.90103**
**D_Bank |**	**1,105**	**2.385485**	**1.391494**	**−6.473036**	**4.652518**
**D_All |**	**1,035**	**2.407192**	**1.438639**	**−6.429156**	**4.958795**
**+**					
**M/R |**	**876**	**1.061829**	**1.129862**	**−6.06905**	**7.132923**

**Source**: own computation.

Real Gross Domestic Product(**RGDP**). It has 1,152 observations with a mean value of 22.92435, a standard deviation of 1.480331, a minimum value of 18.77475, and a maximum value of 27.00616. This suggests that the average real GDP is approximately 22.92, with a relatively low standard deviation indicating that the values are relatively close to the mean. The Bank liquid reserves to bank assets ratio (**R/A**) has 920 observations with a mean of 2.878732, a standard deviation of 0.8249815, a minimum of 0.4937103, and a maximum of 5.966429. This indicates that the average value of this variable is around 2.88, with a relatively high standard deviation suggesting a wide spread of values.

Openness to trade(**OPP**): has 899 observations, with a mean of 0.6994543, a standard deviation of 0.4374 776, a minimum of 0.0631788, and a maximum of 3.650533. Gross domestic product per capita (**GDPD**): with 1,152 observations. The mean is 11.56909, the standard deviation is 2.3975 45, the minimum is 4.526336, and the maximum is 16.58105. Broad Money growth (**M_g**), with 1,089 observations. The mean is 3.232526, the standard deviation is 0.6595083, the minimum is 3.344119, and the maximum is 5.174956. The negative minimum value suggests that this variable can be harmful, indicating a possible deficit or negative value in some cases. Inflation(INF), with 1,018 observations. The mean is 1.820943, the standard deviation is 1.223747, the minimum is 4.696737, and the maximum is 7.874786. GDP deflator with 1,152 observations. The mean is 4.472884; the standard deviation is 1.002485; domestic credit to the private sector by banks (D_Bank) has 1,105 observations. The mean is 2.385485; the standard deviation is 1.391494. Domestic credit to the private sector (D_alll) has 1,035 observations. The mean is 2.407192; the standard deviation is 1.438639, whereas the Broad money-to-total reserves ratio(M/R) is with 876 observations.

These statistics provide a comprehensive overview of the economic health and performance of the countries under consideration, offering insights into various economic indicators and their implications.

### 4.4. Stationarity test

The dataset exhibits unit-root behaviour or remains stationary, in line with [15] recommendations. We utilize the panel extension of Fisher’s Augmented Dickey-Fuller (ADF) test and the Im-Pesaran-Shin test. These tests are suitable for unbalanced panels with fixed effects and time trends, as advised [16]and [17]. The null hypothesis for these tests suggests that all panels are non-stationary, while the alternative hypothesis indicates that some panels are stationary. We reject the null hypothesis of non-stationarity if the p-value is zero; otherwise, we accept it. Our ADF test results (see Appendix 1) indicate that eleven variables—RGDP, OPP, R/A, GDPC, M-G, inf, M, GDPD, D-Bank, D-all, and M/R—are stationary at their levels according to both the Fisher-type test and the Im-Pesaran-Shin unit root test. However, RGDP is stationary with drift and demeaning in the case of ADF. The remaining seven variables also display stationarity. Therefore, there is evidence supporting the possibility that some panels are non-stationary, while we reject the null hypothesis that all panels exhibit non-stationarity. To tackle this issue, we employ the GMM estimation technique.

The coefficients of lagged dependent variables. The outcome indicates that the system GMM model is chosen because its estimates are less than those from the fixed effects, see Table 3 below.

**Table 3:** Model selection process.

Laggeddependent variable	POOL OLs Estimate	Fe coefficient	DGMM coefficient	Remark
lRGDP L1	.999	.96	.840	SGMMis selected

Table 4 provides results for five different estimation models. The Anderson canon correlation LM test and the Sargan test suggest the absence of autocorrelation and the instruments’ validity. The Sargan test’s p-values are enhanced by the inclusion of more variables. Our analysis primarily centres on the whole model, specifically the Mostly Two-Step System GMM, as both it and the one-step GMM were chosen. However, the two-step GMM is widely regarded as more precise and reliable compared to the one-step estimation method [18]

**Table 4:** Five different estimation results Dependent variable: lnRGD.

VARIABLES	MODEL 1	MODEL 2	MODEL 3	MODEL 4	MODEL 5
**LNRGDP**	.9992906**	.837592**	.9637461**	1.00791**	1.008206**
**L1**	(.0020171)	(.0455847)	(.0083681)	(.0090665)	(.0115729)
	0.000	0.000	0.000	0.000	0.000
LNBR/A	.0017984	.0100034*	.0084064	.0207915**	.0199148**
	(.0032864)	(.0053875)	(.007586)	(.0077243)	(.0052803)
	0.584	0.064	0.268	0.007	0.000
**OPP |**	.0024704 (.0052627)	.0423464** (.0081726)	.246799** (.0199027)	.1206666** (.0170465)	.1081628** (.0178259)
	0.639	0.000	0.000	0.000	0.000
**GDPC |**	−.0010386 (.0008492)	−.0009048 (.0009843)	−.001336 (.0008364)	−.0017849 (.0009642)	−.0013149 (.0005803)
	0.221	0.359	0.110	0.64	0.23
**LNM_G |**	.0035127 (.0033369)	.0009415 (.0039644)	−.0005608 (.0044559)	.0035274 .(0046632)	.0051011* (.0021416)
	0.292	0.812	0.900	0.449	0.017
**LNCPI |**	−.0001304 (.0015289)	.0010398 (.001795)	.0004246 (.0016884)	.0011884 (.0019323)	.0015218* (.000754)
	0.932	0.563	0.801	0.539	0.044
**GDPD |**	0031906 (.0021083)	.0003885 (.0027293)	.000594 (.0025035)	.0017148 (.0028736)	0021124* (.0011045)
	0.130	0.887	0.812	0.551	0.056
**LND_BANKL |**	−.0993246 .0839199	−.1812526 .0971456	−.2049723* (.1008573)	−.2501223* (.1126912)	(−.202048) .1064009*
	0.237	−0.063	0.042	0.026	0.058
**LND_ALL |**	−.0139638 (.016531)	−.0181301 (.0367938)	−.2049723* (.1008573)	.0181718 (.0392133)	.0058505 (.0177108)
	0.398	0.622	0.042	0.643	0.741
**LNM/R**	.0034597** (.0017186)	0020779 (.0019147)	.0025995 (.0021827)	0025995 (.0021827)	.0029868** .0008929
	0.044	0.278	0.234	0.234	0.001
**_CONS |.**	0331293 (.0505841) 0.513	4.330118** (.6861115) 0.000	1.48524* (.6352804 0.019	.-.294875 .2077339 0.156	−.2989543 (.2647598) 0.259
Number of observation	466	450	252	1050	800
Number of instruments			277	248	248
P values of ar(1)	0.000	0.000	0.300	0.000	0.000
P-values of Anderson canon. corr.Im test		0.020	0.86	0.0045	0.264
P-values of sargan			0.00	0.000	0.000

Source own computation

* and ** indicate levels of significance at 90% and 95% respectively

Computed using STATA v15, the first row presents the coefficients of the explanatory variables. The second row (in parentheses) indicates the standard deviations. The third row lists the P-values.

The two-step GMM model indicates that actual gross domestic product at lag one(lnRGDPt-1) remains positively significant at a 5 per cent level. Specifically, a 1% increase in the previous year’s gross domestic product leads to a 0.999% increase in the current year’s economic growth. The study reveals that the ratio of bank liquid reserves to bank assets (R/A), serving as an indicator of financial development, has a positive and significant impact on the economic growth of Sub-Saharan Africa (SSA). Specifically, it indicates that for every 1% increase in R/A, the economic development of SSA increases by 0.199. This finding aligns with the notion that a higher ratio of liquid reserves to assets in banks is associated with greater economic growth in the region [19]and [20]. Ensuring sufficient liquid reserves is essential for the financial stability of banks, particularly in the volatile economic conditions prevalent in many Sub-Saharan African (SSA) countries. Such stability supports the creation of a more favourable climate for investment and economic expansion [21].

When Sub-Saharan Africa (SSA) embraces trade openness, it’s like opening doors to new opportunities. Imagine this: for every 1% increase in trade openness, the region’s real GDP grows by about 0.1081628. It’s like for every step they take towards welcoming trade, their economy leaps forward. This isn’t just about numbers, though. It’s about real-life benefits. More trade means SSA countries can invest more in the things that strengthen economies, like roads, bridges, and factories. And it’s not just about the big stuff. It’s also about the everyday things that make life better, like having more choices in the market or finding that special something that

was once hard to get [22]. So, when SSA countries open up to trade, they’re not just boosting their economy on paper. They’re building a more vibrant future for everyone [23].

The findings demonstrate that monetary growth (M_G), another indicator of financial development, has a positive and significant correlation with the growth of Sub-Saharan Africa (SSA). Specifically, a 1% increase in M_G results in a 0.0051011% increase in SSA’s actual gross domestic product (GDP). This suggests that when monetary growth aligns with financial development, it can amplify the impact of financial systems on economic growth, particularly in the context of robust telecommunication, infrastructure, etc. Furthermore, moderate monetary growth can spur investment and consumption by making credit more accessible and affordable, thereby propelling economic growth [24].

The Consumer Price Index (CPI), serving as a proxy for inflation, has a positive and significant impact on the economic growth of Sub-Saharan Africa (SSA). This relationship indicates that a 1% increase in the CPI leads to an actual gross domestic product (GDP) of SSA being affected by a factor of 0.0152. This finding aligns with the research conducted by [25]. However, it’s crucial to recognize that while these effects can temporarily boost economic growth, they require careful management. If inflation expectations become unstable and inflation increases rapidly, it can result in financial instability and a decrease in purchasing power, potentially damaging long-term economic growth. Central banks typically employ monetary policy to stabilize and predict inflation expectations, aiming to maintain economic stability.

The GDP deflator exhibits a positive and significant correlation with the economic growth of Sub-Saharan Africa (SSA), suggesting that a 1% increase in the GDP deflator enhances the economic development of SSA by 0.021124. This finding aligns with the research conducted by [26]

A higher GDP deflator signifies rising price levels, potentially indicating inflation. Moderate inflation is generally linked to healthy economic growth, as it can stimulate consumption and investment. However, if the GDP deflator increases rapidly, it may indicate runaway inflation, which can negatively impact economic stability and growth. High inflation can diminish purchasing power, decrease the real value of money, and introduce economic uncertainty, potentially dampening investment and hindering growth [27].

Broad money to total reserves ratio(**M/R**), the third proxy of financial development has a positive and significant effect on the Economic growth of SSA, which means a 1% increase in M/R, the actual gross domestic product of SSA increases by.0029868. The Broad Money to Total Reserves ratio is an essential financial indicator that reflects the liquidity of a country’s banking system. It measures the proportion of broad money (the total amount of money in circulation plus demand deposits) to the total reserves held by banks. This ratio is crucial for understanding the health of a country’s financial system and its potential impact on economic growth, especially in Sub-Saharan Africa (SSA) [28] and [29].

The domestic credit provided to the private sector by banks (D_bank) has a negative and significant impact on the economic growth of Sub-Saharan Africa (SSA). Specifically, a 1% increase in D_bank discourages the economic development of SSA by 0.202048, aligning with the findings of [30]

This suggests that rapid credit growth can lead to over-indebtedness among businesses and households, potentially resulting in financial crises and economic downturns. Furthermore, excessive credit may cause a misallocation of resources, with funds being directed into unproductive or speculative activities instead of productive investments [31].

## 5. Conclusions and policy implications

The study on financial development and economic growth in Sub-Saharan Africa using System GMM analysis has provided valuable insights into the complex relationship between financial development indicators and economic performance in the region. Based on the empirical findings and discussions presented in the research, the following conclusions and policy implications are forwarded.

The study reveals a positive and significant impact of financial development indicators like bank liquid reserves to bank assets ratio (R/A), trade openness, and the broad money to total reserves ratio (M/R) on economic growth in Sub-Saharan Africa. This underscores the importance of a well-developed financial sector in fostering economic growth and stability. On the other hand, domestic credit provided to the private sector by banks (D_bank) has a negative and significant impact on the economic growth of Sub-Saharan Africa (SSA).

Policymakers in Sub-Saharan Africa should focus on enhancing financial sector development to promote economic growth. This includes improving liquidity, increasing trade openness, and ensuring efficient resource allocation through sound financial intermediation.

Given the rapid growth of financial sectors in the region and existing vulnerabilities, there is a need for robust regulatory frameworks to maintain financial stability. Countercyclical regulations should be implemented to mitigate risks associated with fast credit growth.

### Further research

Future studies should delve deeper into the specific mechanisms through which financial development influences economic growth in Sub-Saharan Africa. Exploring the impact of different financial instruments, regulatory policies, and institutional factors can providemore nuancedinsightsintothisrelationship.

## Supporting information

S1 Appendix Stationarity tests results for the variables used in the analysis. (DOCX)

S1 Data sets Panel dataset of macroeconomic and financial development indicators for Sub-Saharan African countries (1998–2022). (XLSX)

## References

[1] Al-Malkawi HAN, Marashdeh H, Abdullah N. Financial Development and Economic Growth in the UAE: Empirical Assessment Using ARDL Approach to Co-Integrated. International J Economics and Finance. 2012;4:105.

[2] Ustarz Y, Fanta AB. Financial development and economic growth in sub-Saharan Africa: A sectoral perspective. Cogent Economics & Finance. 2021;9(1). doi: 10.1080/23322039.2021.1934976

[3] De Gregorio J, Guidotti PE. Financial development and economic growth. World Development. 1995;23(3):433–48. doi: 10.1016/0305-750x(94)00132-i

[4] Puatwoe JT, Piabuo SM. Financial sector development and economic growth: evidence from Cameroon. Financ Innov. 2017;3(1). doi: 10.1186/s40854-017-0073-x

[5] Blundell R, Bond S. Initial conditions and moment restrictions in dynamic panel data models. Journal of Econometrics. 1998;87(1):115–43. doi: 10.1016/s0304-4076(98)00009-8

[6] King RG, Levine R. Finance and growth: Schumpeter might be right*. Q J Econ. 1993;108(3):717–37. doi: 10.2307/2118406

[7] Aizenman J, Jinjarak Y, Park D. Capital Flows and Economic Growth in the Era of Financial Integration and Crisis. In: 1990. http://www.nber.org/papers/w17502

[8] Levine R, Zervos S. Stock market development and long-run growth. World Bank Econ Rev. 1996;10(2):323–39. doi: 10.1093/wber/10.2.323

[9] Rolervie S. “… and economic growth”. Sci. 1962;2(10):4–7. doi: 10.1002/j.2326-1951.1962.tb00530.x

[10] Bloch H, Tang SHK. The role of economic development in economic growth. Prog Dev Stud. 2003;3(3):243–51. doi: 10.1191/1464993403ps063pr

[11] Guillaumont S, Jeanneney, Kpodar K. No title. Econ prévision. 2006;174(3):87–111.

[12] Oumarou S, Ceylan NB, Kapusuzoglu A. Financial development and economic growth: Evidence from Sub-Saharan Africa. Contributions to Finance and Accounting. 2021. 10.1007/978-3-030-68612-3_5

[13] Piabuo SM, Tieguhong JC. Health expenditure and economic growth - a review of the literature and an analysis between the economic community for central African states (CEMAC) and selected African countries. Health Econ Rev. 2017;7(1):23. doi: 10.1186/s13561-017-0159-1 28593509 PMC5462666

[14] Solow RM. A Contribution to the Theory of Economic Growth. The Quarterly Journal of Economics. 1956;70(1):65. doi: 10.2307/1884513

[15] Salazar DA. Salazar’s Grouping Method: Effects on Students’ Achievement in Integral Calculus. J Educ Pract. 2014;5(15):119–26.

[16] Im KS, Pesaran MH, Shin Y. Testing for unit roots in heterogeneous panels. J Econom. 2003;115(1):53–74. doi: 10.1016/S0304-4076(03)00092-7

[17] Choi I. Unit root tests for panel data. Journal of International Money and Finance. 2001;20(2):249–72. doi: 10.1016/s0261-5606(00)00048-6

[18] Hwang J, Sun Y. Should we go one step further? An accurate comparison of one-step and two-step procedures in a generalized method of moments framework. Journal of Econometrics. 2018;207(2):381–405. doi: 10.1016/j.jeconom.2018.07.006

[19] Asante GN, Takyi PO, Mensah G. The impact of financial development on economic growth in sub-Saharan Africa. Does institutional quality matter?. Dev Stud Res. 2023;10(1). doi: 10.1080/21665095.2022.2156904

[20] Iwayemi A, Fowowe B. Impact of oil price shocks on selected macroeconomic variables in Nigeria. Energy Policy. 2011;39(2):603–12. doi: 10.1016/j.enpol.2010.10.033

[21] Younsi M, Nafla A. Financial stability, monetary policy, and economic growth: Panel data evidence from developed and developing countries. J Knowl Econ. 2019;10(1):238–60. doi: 10.1007/s13132-017-0453-5

[22] Mugun W. Effect of trade openness on economic growth in sub-saharan africa: Dynamic panel analysis. EPRA Int J Econ Bus Manag Stud. 2021;(March):23–35. doi: 10.36713/epra6388

[23] Bunje MY, Abendin S, Wang Y. The Effects of Trade Openness on Economic Growth in Africa. OJBM. 2022;10(02):614–42. doi: 10.4236/ojbm.2022.102035

[24] Junior Abeka M, Andoh E, Gatsi JG, Kawor S. Financial development and economic growth nexus in SSA economies: The moderating role of telecommunication development. Cogent Econ Financ. 2021;9(1). doi: 10.1080/23322039.2020.1862395

[25] Dědeček R, Dudzich V. Exploring the limitations of GDP per capita as an indicator of economic development: a cross-country perspective. Rev Econ Perspect. 2022;22(3):193–217. doi: 10.2478/revamp-2022-0009

[26] Of 26 O, T H E C. The long-range economic assumptions for the 2022 trustees report principal economic assumptions. 2022.

[27] Ofori IK, Obeng CK, Asongu SA. What really drives economic growth in Sub-Saharan Africa? Evidence from the Lasso Regularization and Inferential Techniques. Springer US. 2022;(0123456789). doi: 10.1007/s13132-022-01055-1 PMC9589612 40479378

[28] Haralayya D, Aithal S. Implications of banking sector on economic development in India. 2021;30:1068–80.

[29] Abeka MJ, et al. Financial development and economic growth nexus in SSA economies: The moderating role of telecommunication development. Cogent Econ Financ. 2021;9(1). doi: 10.1080/23322039.2020.1862395

[30] Asante GN, Takyi PO, Mensah G. The impact of financial development on economic growth in sub-Saharan Africa. Does institutional quality matter?. Dev Stud Res. 2023;10(1):2156904. doi: 10.1080/21665095.2022.2156904

[31] Noureddine K. Relationship between domestic credit provided by financial sector and economic growth in Algeria since 1970-2018. 2022.

