# Supplementary material for: Financial development and economic growth in Sub-Saharan Africa using system GMM analysis
Source: PLoS One. 2026 Jun 4;21(6):e0349118. doi: 10.1371/journal.pone.0349118 (PMC13235874; doi:10.1371/journal.pone.0349118)
Supplement: S1 Appendix — (DOCX) [file pone.0349118.s001.docx]

Appendix

Stationarity tests

| Variables | **Im-Pesaran-Shin** unit-root test | | | **Fisher-type** unit-root test | | | |
| --- | --- | --- | --- | --- | --- | --- | --- |
|  | Stat | P-  value | Decision at I(0) | Fisher unit root test with drift | Statistics | P- | Decision at I(0) |
| lnRGDP | 3.144 | 0.999 | Nonstationary | Inverse chi-squared(96) P | 229.09 | 0.000 | Stationary |
| lnGDP  with trend & demeaned | 1.6772 | 0.046 | Stationary | Inverse normal Z | 6.853 | 0.000 |  |
|  |  |  |  | Inverse logit t(244) | 7.263 | 0.000 |  |
|  |  |  |  | Modified inv. Chi-square | 9.603 | 0.000 |  |
| library | 2.7896 | 0.000 | Stationary | Inverse chi-squared(96) P | 309.39 | 0.000 | Stationary |
|  |  |  |  | Invers normal Z | 11.253 | 0.000 |  |
|  |  |  |  | Inverse logit t(244) | 12.287 | 0.000 |  |

|  |  |  |  | Modified inv. Chi- square | 16.352 | 0.000 |  |
| --- | --- | --- | --- | --- | --- | --- | --- |
| OPP | 1.498 | 0.045 | Stationary | Inverse chi- squared(96) P | 214.54 | 0.000 | Stationary |
|  |  |  |  | Invers normal Z | 8.33 | 0.000 |  |
|  |  |  |  | Inverse logit t(244) | 8.53 | 0.000 |  |
|  |  |  |  | Modified inv. Chi square | 10.93 | 0.00 |  |
| GDPC | 16.67 | 0.000 | stationary | Inverse chi- squared(96) P | 667.543 | 0.000 | Stationary |
|  |  |  |  | Invers normal Z | 21.38 | 0.000 |  |
|  |  |  |  | Inverse logit t(244) | 27.281 | 0.00 |  |
|  |  |  |  | Modified inv. Chi- square | 42.181 | 0.000 |  |
| lnMR | 3.598 | 0.000 | Stationary | Inverse chi-squared(96) P | 370.59 | 0.000 | Stationary |
|  |  |  |  | Invers normal Z | 13.612 | 0.000 |  |
|  |  |  |  | Inverse logit t(244) | 14.89 | 0.000 |  |
|  |  |  |  | Modified inv. Chi- square | 20.53 | 0.000 |  |
| lnINF | 9.09 | 0.000 | Stationary | Inverse chi- squared(96) P | 439.763 | 0.000 | Stationary |
|  |  |  |  | Invers normal Z | 15.204 | 0.000 |  |
|  |  |  |  | Inverse logit t(244) | 17.612 | 0.000 |  |
|  |  |  |  | Modified inv. Chi square | 25.63 | 0.000 |  |
| lnMSP | 3.9865 | 0.000 | Stationary | Inverse chi- squared(96) P | 374.93 | 0.000 | Stationary |
|  |  |  |  | Invers normal Z | 12.11 | 0.000 |  |
|  |  |  |  | Inverse logit t(244) | 12.69 | 0.000 |  |
|  |  |  |  | Modified inv. Chi square | 16.65 | 0.000 |  |
| gdpdf | 15.84 | 0.00 | Stationary | Inverse chi- squared(96) P | 651.15 | 0.000 | Stationary |
|  |  |  |  | Invers normal Z | 20.818 | 0.000 |  |
|  |  |  |  | Inverse logit t(244) | 26.499 | 0.000 |  |
|  |  |  |  | Modified inv. Chi square | 41.22 | 0.000 |  |
| lnBS | 3.828 | 0.0001 | Stationary | Inverse chi- squared(96) P | 331.1006 | 0.000 | Stationary |
|  |  |  |  | Invers normal Z | 12.5931 | 0.000 |  |
|  |  |  |  | Inverse logit t(244) | 13.452 | 0.000 |  |
|  |  |  |  | Modified inv. Chi-square | 17.62 | 0.000 |  |
| lnCPS | 2.989 | 0.0014 | Stationary | Inverse chi- squared(96) P | 291.42 | 0.000 | Stationary |
|  |  |  |  | Invers normal Z | 11.19 |  |  |
|  |  |  |  | Inverse logit t(244) | 11.55 |  |  |
|  |  |  |  | Modified inv. Chi square | 14.701 |  |  |
| lnMRR | 0.03567 | 0.0456 | Stationary | Inverse chi- squared(96) P | 261.934 | 0.000 | Stationary |
|  |  |  |  | Invers normal Z | 10.13 |  |  |
|  |  |  |  | Inverse logit t(244) | 11.14 |  |  |
|  |  |  |  | Modified inv. Chi-square | 14.72 |  |  |
